# Inherited and Acquired Decrease in Complement Receptor 1 (CR1) Density on Red Blood Cells Associated with High Levels of Soluble CR1 in Alzheimer’s Disease

**DOI:** 10.3390/ijms19082175

**Published:** 2018-07-25

**Authors:** Rachid Mahmoudi, Sarah Feldman, Aymric Kisserli, Valérie Duret, Thierry Tabary, Laurie-Anne Bertholon, Sarah Badr, Vignon Nonnonhou, Aude Cesar, Antoine Neuraz, Jean Luc Novella, Jacques Henri Max Cohen

**Affiliations:** 1 Department of Internal Medicine and Geriatrics, Reims University Hospitals, Maison Blanche Hospital, 45 rue cognac Jay, 51092 Reims, France; labertholon@chu-reims.fr (L.-A.B.); sbadr@chu-reims.fr (S.B.); vnonnonhou@chu-reims.fr (V.N.); jlnovella@chu-reims.fr (J.L.N.); 2 Faculty of Medicine, University of Reims Champagne-Ardenne, EA 3797, 51092 Reims, France; 3 Institut National de la Santé et de la Recherche Médicale (INSERM), Centre de Recherche des Cordeliers, UMR 1138 Equipe 22, Paris Descartes, Sorbonne Paris Cité University, 75006 Paris, France; sarah.feldman@aphp.fr (S.F.); antoine.neuraz@aphp.fr (A.N.); 4 Department of Medical Informatics, Necker-Enfants Malades Hospital, Assistance Publique des Hôpitaux de Paris (AP-HP), 75015 Paris, France; 5 Department of Immunology, Reims University Hospitals, Robert Debré Hospital, 51092 Reims, France; akisserli@chu-reims.fr (A.K.); vduret@chu-reims.fr (V.D.); ttabary@chu-reims.fr (T.T.); jhmcohen@gmail.com (J.H.M.C.); 6 Faculty of Medicine, University of Reims Champagne-Ardenne, LRN EA 4682, 51092 Reims, France; 7 Department of Research and Innovation, Reims University Hospitals, Robert Debré Hospital, 51092 Reims, France; acesar@chu-reims.fr

**Keywords:** Alzheimer’s disease, complement receptor 1, *CR1* length polymorphism, CR1 density, complement C3b/C4b receptor, complement, dementia, molecular biology, neurosciences, genetic risk

## Abstract

The complement receptor 1 (*CR1*) gene was shown to be involved in Alzheimer’s disease (AD). We previously showed that AD is associated with low density of the long CR1 isoform, CR1*2 (S). Here, we correlated phenotype data (CR1 density per erythrocyte (CR1/E), blood soluble CR1 (sCR1)) with genetic data (density/length polymorphisms) in AD patients and healthy controls. CR1/E was enumerated using flow cytometry, while sCR1 was quantified by ELISA. *CR1* polymorphisms were assessed using restriction fragment length polymorphism (RFLP), pyrosequencing, and high-resolution melting PCR. In AD patients carrying the H allele (*Hin*dIII polymorphism) or the Q allele (Q981H polymorphism), CR1/E was significantly lower when compared with controls carrying the same alleles (*p* < 0.01), contrary to sCR1, which was significantly higher (*p* < 0.001). Using multivariate analysis, a reduction of 6.68 units in density was associated with an increase of 1% in methylation of *CR1* (estimate −6.68; 95% confidence intervals (CIs) −12.37, −0.99; *p* = 0.02). Our data show that, in addition to inherited genetic factors, low density of CR1/E is also acquired. The involvement of CR1 in the pathogenesis of AD might be linked to insufficient clearance of amyloid deposits. These findings may open perspectives for new therapeutic strategies in AD.

## 1. Introduction

Alzheimer’s disease (AD) is a neurodegenerative disease that depends on both genetic and environmental factors. Genetic studies showed that the determinants of AD are manifold. In fact, while certain early-onset forms of AD are directly linked to mutations in genes that follow traditional Mendelian transmission, it was also established that other genetic risk factors play a role in sporadic forms of the disease. Genome-wide association studies (GWASs) identified variations in over 20 loci that contribute to disease risk, including the complement component (C3b/C4b) receptor 1 (*CR1*) gene [1,2,3,4,5,6,7].

The *CR1* gene encodes the complement receptor 1 (*CR1*), which is one of the regulators of complement activity. CR1 is a membrane-bound glycoprotein that binds to the complement proteins C3b, C4b, C3bi, and C1q. In humans, 90% of the total circulating CR1 is found on erythrocytes [8]. On the surface of erythrocytes, CR1 binds to C3b- or C4b-opsonized microorganisms or immune complexes, thus facilitating their clearance from circulation [9,10,11]. By limiting the deposition of C3b and C4b, CR1 might prevent excessive complement activation. In this way, the presence of CR1 on erythrocytes is viewed as a critical component in protecting tissues against immune-complex deposition and subsequent disease, such as AD [12,13].

CR1 is a glycoprotein of approximately 200 kDa. The extracellular domain of the most common form of CR1 is composed of a series of 30 repeating units named short consensus repeats (SCRs). The SCRs are arranged in tandem groups of seven, known as long homologous regions (LHRs). CR1 is arranged in four LHRs designated as LHR-A, -B, -C, and -D, arising from duplication of a seven-SCR unit [14,15].

CR1 presents three types of polymorphisms: density polymorphism, structural polymorphism (length), and Knops blood group polymorphism [14,16].

The density polymorphism is a stable phenotype that accounts for the constitutive expression level of CR1 on erythrocytes, although acquired deficiency may also occur in some diseases, such as systemic lupus erythematosus (SLE) and acquired immune deficiency syndrome (AIDS) [17]. In Caucasians, erythrocytes from different healthy subjects show up to a 10-fold variation in the number of CR1 molecules per erythrocyte (range: 150–1200 molecules/erythrocyte) [18]. Moreover, previously published data showed that CR1 density was not correlated with age [19,20]. CR1 density on erythrocytes is genetically associated with an autosomal co-dominant bi-allelic system on the *CR1* gene, which is correlated with a *Hin*dIII restriction fragment length polymorphism (RFLP) [21]. A single-point mutation in intron 27 of the *CR1* gene, which is located between the exons that encode the second SCR in LHR-D, results in the generation of a polymorphic *Hin*dIII site within this region [22]. Genomic *Hin*dIII fragments of 7.4 and 6.9 kDa identify alleles associated with high (H allele) or low (L allele) CR1 density, respectively, on erythrocytes [14,16,21]. CR1 density on erythrocytes is also associated with the presence of a nucleotide mutation (G3093T) in exon 19 encoding the polymorphism Q981H in SCR 16 (LHR-C) of *CR1*. CR1 density is higher in individuals who are homozygous for the QQ genotype, and lower in individuals who are homozygous for the HH genotype [15,23,24].

The second CR1 polymorphism is the structural polymorphism (length), related to a variation in LHR number. The most common isoform of *CR1* (CR1*1, also termed F), found in about 87% of Caucasians, is composed of four LHRs. The second most common isoform (CR1*2, also termed S), which is found in about 11% of Caucasians, is composed of five LHRs; thus, this isoform contains additional C3b/C4b sites. The two other, rarer, *CR1* isoforms, CR1*3 (F’) and CR1*4 (D), exhibit a deletion of one LHR or the presence of two additional LHRs, respectively [14].

The third CR1 polymorphism is the Knops (KN) polymorphism, whose role in AD remains to be determined [25].

Finally, CR1 is also present in circulation in a soluble form (sCR1) [26], resulting from either the proteolysis of the membrane-bound form of CR1 [27] or exocytosis from erythrocytes (E) [28]. It is hypothesized that sCR1 is a locally active molecule that seems to have highly efficacious complement regulatory and anti-inflammatory activities [16,29]. In fact, sCR1 is a potent local inhibitor that functions in the complement pathways [30]. In addition, increased plasma levels of sCR1 were reported in some autoimmune diseases, such as SLE and glomerulonephritis [31]. In AD, a slight increase in sCR1 was reported in subjects with risk of AD single-nucleotide polymorphisms (SNPs) [32].

The complement’s role in AD pathogenesis was highlighted in different studies [33,34], suggesting that AD is associated with increased complement activation [35,36]. Previous studies showed that the AD risk associated with *CR1* can be explained by low density [20] of the long *CR1* isoform, CR1*2 (S) [35,36,37]. However, the mechanisms underlying the decrease in CR1 density in AD remain to be elucidated. In the current study, we aimed to correlate genetic data (density/length polymorphisms) with phenotypic data (CR1 density per erythrocyte (CR1/E) and soluble CR1 (sCR1)) in patients with AD and control subjects.

## 2. Results

A total of 187 Caucasian subjects (100 AD patients and 87 controls) were investigated. Their main socio-demographic and clinical characteristics are shown in Table 1.

The average age was 81.5 ± 7.2 years for AD patients, and 74.3 ± 6.3 years for controls. Indeed, there was no correlation between age and *CR1* density, using the Pearson correlation coefficient (r = −0.1, *p*= 0.17 in the overall population; r = 0.03, *p* = 0.75 in AD patients; and r = −0.07, *p* = 0.53 in controls).

Moreover, as expected, the *APOE*-ε4 allele, a high level of dependence, and cognitive disorders were associated with AD in our population (*p* = 0.0071, *p* < 10^−4^, and *p* < 10^−4^, respectively). However, no significant differences in sex (*p* = 0.23), place of residence (*p* = 0.73), or comorbidities (*p* = 0.27) were observed between AD patients and controls, which confirmed that the rationale of this study was valid.

CR1/E density in the overall study population was, on average, 677 ± 288. The average CR1 density among AD patients was significantly lower compared to controls (626 ± 272 vs. 737 ± 297; *p* = 0.009). After adjustment for age, this difference remained statistically significant (β = −106.6 ± 47.4; *p* = 0.03).

### 2.1. Association between the Genetic CR1 Density Polymorphism and the CR1 Density Phenotypic Polymorphism

#### 2.1.1. Association between the Genetic *CR1* Density Polymorphism, *Hin*dIII, and the CR1 Density Phenotypic Polymorphism

Among the 187 subjects investigated, 114 exhibited the HH genotype, 65 exhibited the HL genotype, and eight exhibited the LL genotype (Table 2).

**Figure 1:**
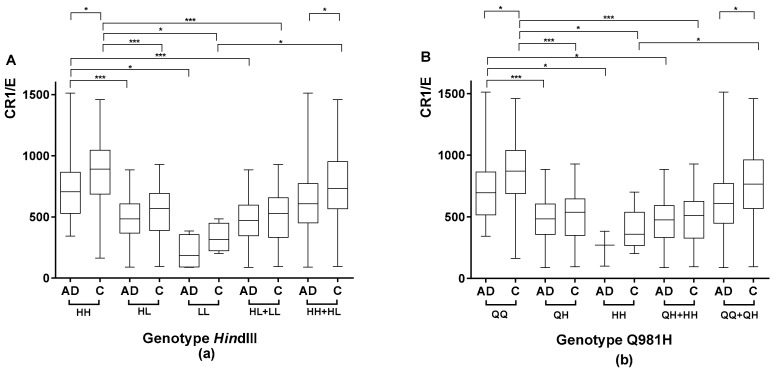
Comparison of the mean number of complement receptor 1 per erythrocyte (CR1/E) according to *CR1* density polymorphisms in Alzheimer’s disease (AD) patients and/or controls. Box plots of CR1/E are shown. The upper and lower limits of the boxes, and the middle line across the boxes indicate the 75th and 25th percentiles, and the median, respectively. The upper and lower horizontal bars indicate the maximum and minimum values, respectively. Wilcoxon’s rank test was used to compare CR1 density among AD patients or among controls according to genotype for non-normally distributed variables. The Mann-Whitney *U* test was used to compare AD patients with controls according to genotype for non-normally distributed variables, and the Student’s *t*-test was used for normally distributed variables; * *p* < 0.01, ** *p* < 0.001, and *** *p* < 0.0001. (**a**) Comparison of the mean number of CR1/E according to *Hin*dIII polymorphisms in AD patients and/or controls. (**b**) Comparison of the mean number of CR1/E according to Q981H polymorphisms in AD patients and/or controls.

Among the AD patients, subjects with the HH genotype had a higher CR1 density (742 ± 262) than subjects carrying the L allele (HL genotype, 486 ± 175, *p* < 0.0001; LL genotype, 210 ± 142, *p* < 0.01), including both the HL and LL genotypes (460 ± 190, *p* < 0.0001), as shown in Figure 1a.

Similar findings were observed in the control subjects (Figure 1a), showing that the genetic criteria were in concordance with the phenotypic criteria (the H allele was associated with a higher density compared to the L allele).

#### 2.1.2. Association between the *CR1* Density Genetic Polymorphism Encoding Q981H and the CR1 Density Phenotypic Polymorphism

Among the 187 subjects investigated, 118 exhibited the QQ genotype, 60 exhibited the QH genotype, and nine exhibited the HH genotype (Table 2).

Among AD patients, subjects with the QQ genotype had a significantly higher CR1 density (730 ± 262) than subjects carrying the H allele (QH genotype, 474 ± 189, *p* < 0.0001; HH genotype, 251 ± 143, *p* < 0.01), including both the QH and HH genotypes (456 ± 194, *p* < 0.01), as shown in Figure 1b.

Similar findings were observed in the control subjects, again showing that the genetic criteria were in agreement with the phenotypic criteria (the Q allele being associated with a higher density compared to the H allele).

#### 2.1.3. Study of the Agreement between the *Hin*dIII Genotype and Q981H

Analysis of the agreement between the *Hin*dIII genotype and Q981H showed excellent results for AD patients (weighted Kappa coefficient: 0.93; 95% confidence intervals (CIs): 0.86, 1.0), control subjects (weighted Kappa coefficient: 0.90; 95% CIs: 0.80, 1.0), and the overall population (AD patients + controls; weighted Kappa coefficient: 0.91; 95% CIs: 0.85, 0.97).

#### 2.1.4. Comparison of CR1 Density Using *Hin*dIII and Q981H Genotype in AD Patients vs. Controls

The comparison of CR1 density between patients and controls according to the *Hin*dIII genotype showed a lower density in AD patients homozygous for the H allele compared with controls homozygous for the H allele (742 ± 262 vs. 864 ± 268, respectively; *p* < 0.01). Furthermore, when combining HH and HL subjects, the average CR1 density was significantly lower in AD patients vs. controls (643 ± 263 and 756 ± 289, respectively; *p* < 0.01), as shown in Figure 1a.

A comparison of CR1 density between AD patients and control subjects according to the Q981H density polymorphism showed that density was significantly lower in patients homozygous for the Q allele compared with controls homozygous for the Q allele (730 ± 262 vs. 866 ± 261, respectively; *p* < 0.01). When combining QQ and QH subjects, the average CR1 density was also significantly lower in AD patients vs. controls (638 ± 267 and 762 ± 289, respectively; *p* < 0.001), as shown in Figure 1b.

These findings suggest that, in addition to genetic factors, the low-density phenotype is acquired in AD.

### 2.2. Evaluation of the CR1 Length Polymorphisms

Table 2 presents the distribution of subjects according to the *CR1* length polymorphism.

### 2.3. Evaluation of the Serum Levels of sCR1

The average level of serum sCR1 in the overall population enrolled in this study was 27.17 ± 21.55 ng/mL. In AD patients, sCR1 levels were significantly higher than in controls (31.60 ± 22.86 vs. 21.96 ± 18.71 ng/mL, respectively; *p* = 0.002). The difference remained significant after adjustment for age (β = 9.3 ± 3.53; *p* = 0.009).

#### 2.3.1. Serum sCR1 Levels According to *CR1* Length Polymorphisms

**Figure 2:**
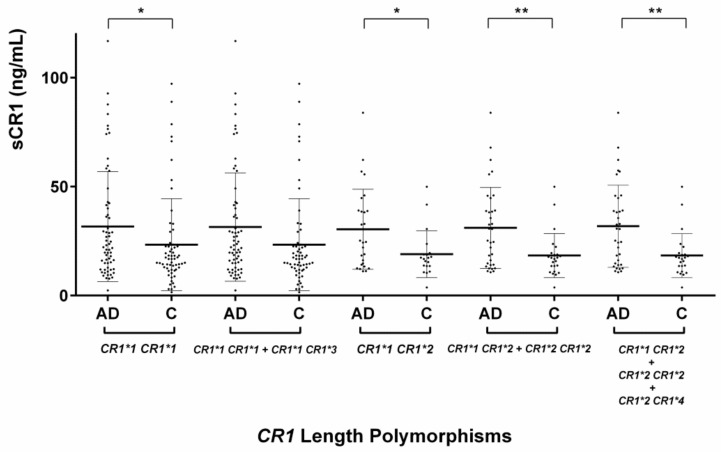
Comparison of soluble complement receptor 1 (sCR1) levels according to *CR1* length polymorphisms in Alzheimer’s disease (AD) patients and controls. The middle line indicates the mean value, and the upper and lower horizontal bars indicate the standard deviation values. A Student’s *t*-test was used to compare AD patients with controls according to *CR1* length polymorphisms for normally distributed variables; * *p* < 0.05, ** *p* < 0.01.

In subjects with the genotype CR1*1 CR1*1, the level of sCR1 was significantly higher in AD patients than in controls (31.69 ± 25.24 vs. 23.38 ± 21.06 ng/mL, respectively; *p* = 0.048). However, when subjects with the *CR1* short alleles (CR1*1 CR1*1 and CR1*1 CR1*3) were pooled, the difference between patients and controls was no longer significant. In subjects with the long *CR1* allele (CR1*1 CR1*2 and CR1*2 CR1*2), the level of sCR1 was significantly higher in AD patients compared to controls (31.08 ± 18.60 vs. 18.35 ± 10.14 ng/mL, respectively; *p* = 0.006). We observed similar findings in heterozygous subjects (CR1*2 CR1*2 subjects), with a significantly higher level of sCR1 in AD patients vs. controls (30.43 ± 18.37 vs. 19.01 ± 10.74 ng/mL, respectively; *p* = 0.027), as shown in Figure 2.

#### 2.3.2. Evaluation of Serum sCR1 Levels According to the *CR1* Density Polymorphisms *Hin*dIII and Q981H

**Figure 3:**
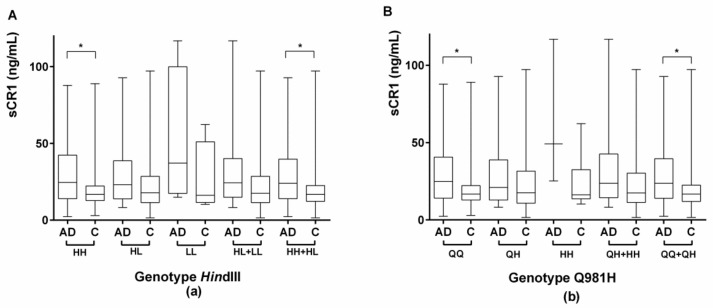
Comparison of sCR1 levels according to *CR1* density polymorphisms, *Hin*dIII and Q981H (genotype), among AD patients and controls. Box plots of sCR1 are shown. The upper and lower limits of the boxes, and the middle line across the boxes indicate the 75th and 25th percentiles, and the median, respectively. The upper and lower horizontal bars indicate the maximum and minimum values, respectively. A Student’s *t*-test was used to compare AD patients with controls according to *CR1* density polymorphisms for normally distributed variables; * *p* < 0.005; sCR1 = soluble CR1. (**a**) Comparison of sCR1 levels according to *Hin*dIII polymorphisms in AD patients and controls. HH = individuals homozygous for the H allele (*Hin*dIII polymorphism); HL = individuals heterozygous for the *Hin*dIII polymorphism; LL = individuals homozygous for the L allele (*Hin*dIII polymorphism). (**b**) Comparison of sCR1 levels according to Q981H polymorphisms in AD patients and controls. QQ = individuals homozygous for the Q allele (Q981H polymorphism); QH = individuals heterozygous for the Q981H polymorphism; HH = individuals homozygous for the H allele (Q981H polymorphism).

There was no significant difference in sCR1 levels according to genotype, either in the overall population or in the AD and control groups separately. The comparison of the levels of sCR1 between AD subjects and controls according to the *Hin*dIII genotype showed that there was a significantly higher level of sCR1 in AD patients homozygous for the H allele compared to controls (31.67 ± 22.23 vs. 20.65 ± 16.99, respectively; *p* = 0.004). sCR1 levels were also significantly higher in AD patients compared to controls when we pooled subjects who were homozygous for the H allele and subjects who were heterozygous for the allele (HH and HL subjects, respectively; 30.77 ± 21.46 and 21.74 ± 18.57, respectively; *p* = 0.003), as shown in Figure 3a.

The comparison of sCR1 levels between AD patients and controls according to the Q981H genotypes showed significantly higher levels in patients homozygous for the Q allele vs. controls (31.44 ± 21.71 vs. 20.65 ± 16.83 ng/mL, respectively; *p* = 0.0038). sCR1 levels were also significantly higher in patients compared to controls when we grouped subjects who were homozygous for the Q allele and subjects who were heterozygous for the allele (QQ and QH subjects, respectively; 30.61 ± 21.41 and 21.82 ± 18.79 ng/mL, respectively; *p* = 0.0048), as shown in Figure 3b.

### 2.4. Association between CR1/E and sCR1 and the Stage of AD

The comparison of CR1/E between AD subjects according to the stage of AD, as assessed by Mini-Mental State Examination (MMSE) scores, showed that CR1/E was significantly lower in patients with moderate or severe AD as compared with mild AD (583.67 ± 238.58 vs. 698.75 ± 295.32, respectively; *p* = 0.034), as shown in Figure S1a. In contrast, there was no significant difference in sCR1 in AD subjects according to the stage of AD (Figure S1b).

In addition, the association between CR1/E and the severity of AD, measured by the MMSE score and tested using univariate linear regression (with MMSE scores alternatively used as a quantitative variable and a categorical variable in two classes) showed that CR1/E density was significantly lower for moderate and severe AD patients than for mild AD patients (estimate: −115.08; 95% CIs: −221.78, −8.39; *p* = 0.035), as shown in Table S1. In contrast, no association was found between sCR1 and AD severity (estimate: −1.12; 95% CIs: −10.45, −8.21; *p* = 0.812), as shown in Table S2.

### 2.5. CR1 Methylation

The second methylation site was associated with a reduction of 6.68 units in density, for an increase of 1% in methylation (estimate: −6.68; 95% CIs: −12.37, −0.99; *p* = 0.02), independently of AD, age, and density polymorphism. Density decreased with age (estimate: −6.51; 95% CIs: −11.79, −1.23; *p* = 0.016), as shown in Table 3.

### 2.6. Assessment of Factors Associated with AD

A multivariate analysis identified six factors that were independently related to AD: age, female sex, *APOE*-ε4 carrier, number of CR1 antigenic sites per erythrocyte (density), the level of sCR1, and the density polymorphism Q981H (Table 4).

We failed to show a significant interaction between *CR1* length polymorphisms and *APOE* (*p* = 0.106), or between CR1 density and *APOE* (*p* = 0.3795).

To investigate the risk associated with different quantitative variations in the explanatory variables, the results of the multivariate analysis presented in Table 4 modeled the adjusted risk for a range of different variations in the two quantitative variables (density and level of sCR1). This enabled us to explore variations that could be clinically relevant, given the absence of data in the literature. Regarding the variable “density”, since the threshold of biological detection is 30 sites and the average density in the general population is 500 (150–1500), according to our multivariate model, a variation of 30 sites was associated with a 6.4% reduction in the risk of developing AD. However, a variation of 30 sites is neither clinically nor biologically relevant. Conversely, a variation of 200 antigenic sites has greater biological discrimination and, according to our model, it was associated with a 35.9% reduction in the risk of developing AD, which is also more clinically relevant. Furthermore, our multivariate model showed that an increase of 20 ng/mL in serum sCR1 was associated with a 1.8-fold increase in the risk of AD (95% CIs: 1.29, 2.84; Table 4).

The multivariate model fitted the data well (*p*-value for the Hosmer and Lemeshow test = 0.23).

## 3. Discussion

The originality of our study resides in the combination of phenotypic and genotypic data relating to *CR1* polymorphisms in a well-characterized cohort of AD patients and control subjects. Furthermore, we confirmed findings from previous works showing that AD is associated with the long *CR1* isoform [35,36], and that low CR1 density could explain the association between *CR1* and AD [20], as identified by GWAS [3].

The present study established that abnormally low CR1 density on erythrocytes was associated with AD, independently of genetic factors. The agreement between the two genotypes associated with the *CR1* density polymorphism (*Hin*dIII and Q981H) was excellent, both in patients and controls. Univariate analysis showed that the presence of the H allele (*Hin*dIII) or the Q allele (Q981H) was associated with significantly higher CR1 density as compared with the L allele (*Hin*dIII) or the H allele (Q981H), both in patients and in controls. However, CR1 density was lower in AD patients compared with controls in the presence of both the H allele (*Hin*dIII) and the Q allele (Q981H). This observation of lower density in carriers of the high-density allele (H for *Hin*dIII or Q for Q981H) was more pronounced in patients who were homozygous for the allele coding for high density. Altogether, our findings suggest that genetic factors determining CR1 density are indeed present; however, non-genetic factors, such as acquired factors, can be involved in AD, resulting in a CR1 low-density phenotype acquired during the course of AD. This might also contribute to AD development.

With regards to the *CR1* length polymorphism, our results generally followed the same trends as those observed in our previous work [20]. No association between length polymorphism-associated genotype and density polymorphism-associated genotype was observed. Conversely, we previously reported an association between the length polymorphism and the density polymorphism at the protein level. Again, these results support our hypothesis that, in addition to constitutive genetic factors such as the long *CR1* allele, which appears to be linked to regulatory factors probably in the promoter region, other non-genetic factors also have an effect on CR1 expression. This may lead to a lower-density phenotype than that expected from the genotype of a given patient, acquired during the course of the disease. Reciprocally, this could result in a decline in the clearance of the amyloid beta 1-42 (Aβ_1-42_) peptide, as well as in a lower control of in situ inflammation, in turn leading to a higher risk of developing AD [13,35,36].

In this study, analysis of five methylation sites revealed an increase in methylation at the second site located in the additional segment of the long *CR1* allele (CR1*2 (S)) suggesting that the increase in methylation of the long *CR1* allele (CR1*2 (S)) might be the direct mechanism of the lower expression of that isoform at the protein level.

In the present study, serum levels of sCR1 were assessed. Firstly, we showed that sCR1 levels were higher in AD patients compared with controls. This could be explained by increased proteolysis of CR1, as shown in patients with diseases related to protease production [17,38], and/or its vesiculation (exocytosis) demonstrated in erythrocytes [28]. As described in SLE, this suggests that, at the peripheral level and during the binding or capture of amyloid peptides, C3b molecules are deposited at the cell surface, and removed together with CR1 as the surrounding molecule via vesiculation of the membrane [39]. Accordingly, the CR1-enriched vesicles are taken into account in sCR1 dosages [40]. Secondly, the univariate analysis performed in our study showed that the long *CR1* alleles (CR1*2 and CR1*4) were associated with higher levels of sCR1 in AD patients compared with controls. In this regard, our results may help explain the pathological observations of Hazrati and colleagues [36], who found that the distribution of CR1 in the brain was different between *CR1* length polymorphism-associated genotypes, hypothesizing that the CR1*1 isoform is transported between protein-sorting compartments, whereas the longer CR1*2 isoform accumulates in the membrane of cytoplasmic vesicles [36]. Taken together, our results suggest that the long *CR1* alleles are linked, during the course of AD, to lower CR1 density, probably due to the effect of other genetic or acquired factors, which might partially explain the increase in sCR1 through proteolysis and/or exocytosis.

Lastly, using multivariate analysis, we identified six factors that were independently associated with AD, namely age, female sex, *APOE*-ε4 carrier, CR1 density, serum sCR1 level, and the density polymorphism Q981H (Q allele). In fact, age, female sex, and the *APOE*-ε4 allele were already described in the literature as risk factors for AD [41]; however, this is the first study showing that CR1 density, serum sCR1 levels, and the density polymorphism Q981H (Q allele) are independent factors related to AD. According to our multivariate model, an increase of 200 CR1 antigenic sites was associated with a 35.9% reduction in the risk of developing AD. This does not imply that subjects with a low density (e.g., 100 CR1/E sites) are at higher risk of developing AD, since the risk of developing AD was not higher in a subject who expressed 200 CR1/E sites than in a subject who expressed 900 CR1/E sites, for example. This can likely be explained by the existence of other factors belonging to other systems. However, when an individual is genetically programmed to display 900 CR1/E sites, yet, under the influence of external factors, expresses a reduced CR1 density of 200 CR1/E, then the risk of developing AD would be increased. Our findings showed a high frequency of the QQ or HH genotype, both in AD patients and in control subjects, which was in line with previous reports [42]. Taken together, our phenotype and genotype findings suggest that the biological pathway of CR1 expression is ruled by both genetic and acquired factors that are intrinsically linked. Thus, an acquired reduction in CR1 density, as opposed to a low-density genotype, seems to be associated with an increased risk of AD. Another finding of our multivariate analysis was that the serum sCR1 level was associated with AD, independently of age, CR1 density, and density polymorphism (Q allele for Q981H).

Our findings suggest that, in addition to genetic factors, a low density of CR1 is also acquired during the course of AD, and that the involvement of CR1 in the pathogenesis of AD might be linked to both insufficient in situ inhibition of complement and/or inflammation, or impaired amyloid protein clearance in the peripheral blood. In fact, an improved understanding of the pathophysiological mechanisms of AD may pave the way toward new therapeutic avenues for this disease. In light of our results, and in view of the physiological role and potential implication of CR1 in the pathogenesis of AD, two avenues deserve to be further explored: the increase in CR1 expression (which requires a better understanding of regulatory factors), and the use of recombinant forms of sCR1 to restore improved control of complement-induced inflammation.

## 4. Materials and Methods

### 4.1. Study Population

The study was approved by the regional ethics committee, and written informed consent was obtained from each participant. All AD patients met the diagnostic criteria for probable AD according to the Diagnostic and Statistical Manual of Mental Disorders, fourth edition (DSM IV) [43], and the criteria of the National Institute of Neurological and Communicative Disorders and Stroke and the Alzheimer’s Disease and Related Disorders Association (NINCDS-ADRDA) [44]. The controls were subjects without any organic brain or cognitive disorders. Subjects with diseases that were likely to affect the physiology of CR1, such as hemolytic anemia, terminal renal or liver failure, or SLE, were excluded from the study. We also excluded subjects receiving treatments likely to modify sCR1 rates, such as non-steroidal or steroidal anti-inflammatory drugs, as well as those receiving treatments likely to modify CR1/E density, such as blood transfusion.

Blood samples were drawn into Vacutainer tubes containing 0.12 mL of 0.15% ethylenediaminetetraacetic acid (EDTA) and 5-mL Vacutainer dry tubes.

### 4.2. Quantification of CR1 Density Using Flow Cytometry

The mean CR1 density on erythrocytes was determined using flow cytometry and a J3D3 monoclonal antibody (moAb) [45], as previously described [46]. Moreover, the anti-CR1 moAb J3B11, and the TO5 and E11 moAbs were also used in flow cytometry or in control experiments [20]. A standard curve was obtained from donors of known CR1 antigenic sites, with a density ranging from 180 to 1000 sites per erythrocyte. Flow cytometry was performed on stained cells using a flow cytometer (FACScan; Becton Dickinson, Mountain View, CA, USA). At least 10,000 events were collected for each sample. The mean fluorescence intensity channel was used to quantify the staining of each sample. The detection threshold was 30 CR1 antigenic sites per erythrocyte.

### 4.3. DNA Extraction

DNA from 2-mL whole blood samples was isolated using the QuickGene-610L (Fujifilm, Asnières, France). The manufacturer’s instructions were followed according to the recommended protocol. Briefly, tubes containing 2 mL of EDTA whole blood, 2.5 mL of lysis buffer (containing guanidine hydrochloride), and 300 μL of proteinase K were mixed and incubated for 5 min at 56 °C. After this incubation, 2.5 mL of ethanol (>99%) was added and mixed, and the samples were applied to the Quickgene Cartridge. Cartridges were placed on the instrument and QuickGene-610L’s pre-programmed protocol automated the rest of the process, before DNA was finally eluted in 500 μL of elution buffer.

#### 4.3.1. *APOE* Genotyping using Amplification and High-Resolution Melting Analyses

PCR was performed in 10-µL volumes in a LightCycler 480 (Roche) using 96-well plates. The Type-it HRM PCR Kit (Quiagen, Courtaboeuf, France) was used following the LightCycler 480 manufacturer’s instructions and specific primer sets (Spot-to-Lab. Montpellier France) for each SNP genotyping rs429358 and rs7412 (primer sequences are available on request from the manufacturer). The temperature-cycling protocol included an initial denaturation step at 95 °C for 10 min, followed by 50 cycles of denaturation at 95 °C for 15 s, annealing for 15 s, and extension at 72 °C with a transition from annealing to extension of 2.2 °C/s. The touchdown program of the annealing step was used with a starting temperature of 65 °C and a progressive decrease of 0.5 °C per cycle to reach the final temperature of 55 °C. The reactions were monitored during PCR at the end of each extension phase. Following the amplification phase, the samples were heated momentarily in the LightCycler to 94 °C for 1 min and rapidly cooled to 40 °C to create heteroduplexes. The melting curves were obtained by heating from 65 °C to 95 °C at 0.02 °C/s and 25 fluorescent acquisitions per °C. High-resolution melting data were analyzed using the gene scanning module of the LightCycler 480 Software. Allele identification was determined using the combination of rs429358 and rs7412 genotyping results.

#### 4.3.2. Assessment of the *CR1* Density Genetic Polymorphism Using *Hin*dIII RFLP

The *CR1* density polymorphism on erythrocytes was determined using PCR amplification and *Hin*dIII restriction enzyme digestion, as described previously [18]. The PCR primers used were 5′–CCTTCAATGGAATGGTGCAT–3′ and 5′–CCCTTGTAAGGCAAGTCTGG–3′. PCR was performed on a MyCycler apparatus using the following conditions: a final volume of 100 µL containing 2 µL of DNA solution (approximately 100–250 ng/µL), a 200-µM concentration of each deoxynucleoside triphosphate, a 0.5-mM concentration of each primer, 2.5 mM MgCl_2_, and 2.5 U of Taq GOLD DNA polymerase (Perkin Elmer Cetus) in the buffer supplied by the manufacturer. The amplification conditions were as follows: 10 min at 94 °C, followed by 40 cycles of 1 min at 94 °C, 1 min at 61 °C, and 2 min at 72 °C, before being held for 10 min at 72 °C.

For RFLP determination, 30 µL of PCR product and 2 µL of *Hin*dIII were incubated in a final volume of 50 µL in the buffer supplied by the manufacturer at 37 °C for 2 h, followed by analysis on a 2% ethidium bromide gel.

Using this protocol, *Hin*dIII digestion did not alter the PCR product (1.8 kb) from individuals who were homozygous for the *CR1* high-density allele (HH). The 1.8-kb band was fully split into two smaller bands of 1.3 and 0.5 kb in samples from individuals who were homozygous for the *CR1* low-density allele (LL).

#### 4.3.3. Determination of the *CR1* Density Genetic Polymorphism by Pyrosequencing

##### Q981H (Exon 19) Amplification for Pyrosequencing

The PCR primers used were 16aL 5′–GCTACATGCAGGTTGAGACCTTAC–3′ and PCRE111926RE 5′–CTGAGATGTGGCTAGAAAGTAC–3′. PCR was performed on a MyCycler apparatus using the following conditions: 50 µL of final volume containing 1 µL of DNA solution (approximately 100–250 ng/µL), a 200-mM concentration of each deoxynucleoside triphosphate, a 0.5-mM concentration of each primer, 2 mM MgCl_2_, and 1.25 U of Taq DNA polymerase (Promega, Madison, WI, USA) in the buffer supplied by the manufacturer. The amplification conditions were as follows: 10 min at 94 °C, followed by 40 cycles of 1 min at 94 °C, 1 min at 64 °C, and 1 min at 72 °C, before being held for 10 min at 72 °C. Nested PCR was then performed using the primers PCRE111926RE and PCRssCR1LikeBIOT 5′–AAATCATGTAAAACTCCTCCAGA–3′ biotinylated on its 5′ end to allow immobilization of the PCR product on streptavidin beads and the preparation of single-stranded DNA. One microliter of the first PCR reaction diluted at 1:500 in water was used for the nested PCR. The procedure used for the second PCR procedure was the same as that used in the first PCR. All fragments were subjected to gel electrophoresis on agarose gels containing ethidium bromide, before isolation for pyrosequencing.

##### Pyrosequencing

Primers were drawn to anneal adjacent to codons Q981H of the *CR1* gene: PCR2Q981H35rev 5′–TGATTCTGGATCCAA–3′. The biotinylated PCR product (40 µL) was immobilized onto 4 µL of streptavidin-coated Sepharose beads (>1.2 nmol binding capacity; Amersham Pharmacia Biotech, Uppsala, Sweden) in 40 µL of binding buffer (10 mM Tris-HCl (pH 7.6), 2 M NaCl, 1 mM EDTA, and 0.1% Tween 20) on a shaker (1400 rpm) at room temperature for 10 min. PCR products immobilized on beads were transferred to a 96-well filter plate (Millipore, Molsheim, France) and vacuum-dried. Single-stranded DNA was obtained by adding 50 µL of denaturation solution (0.2 M NaOH) for 1 min. The immobilized strand was washed twice with 150 µL of washing buffer (10 mM Tris-acetate (pH 7.6)), re-suspended in 45 µL of annealing buffer (20 mM Tris-acetate (pH 7.6) and 2 mM magnesium acetate), and transferred into wells containing 15 pmol of sequencing primer in a volume of 1.5 µL of annealing buffer. The plate was heated at 61 °C for 5 min. Real-time pyrosequencing was performed at 28 °C in an automated 96-well pyrosequencer using PSQ SNP 96, with enzymes and substrate (Pyrosequencing AB, Uppsala, Sweden), with cyclic dispensation of nucleotides. The computer analysis was based on an algorithm that compared the height of the different peaks and the base number of the polymorphic fragment.

#### 4.3.4. Determination of the rate of *CR1* methylation by Pyrosequencing

##### Amplification of the *CR1* gene (LHR-B segment and LHR-C segment) for Pyrosequencing

The PCR primers used were methF1 (5′–GGAAGTTGATGAGGTATGTATAGTATAA–3′) and methR1biot (5′–AATACCATTTCCAAAAAAAATAAAATCCA–3′). PCR was performed on a MyCycler apparatus using the following conditions: 50 µL of final volume containing 1 µL of DNA solution (approximately 100–150 ng/ µL), a 16-nM concentration of each deoxynucleoside triphosphate, a 0.5-mM concentration of each primer, 1.5 mM MgCl_2_, and 2 U of AmpliTaq Gold DNA polymerase (Perkin-Elmer, Roissy, France) in the buffer supplied by the manufacturer. The amplification conditions were as follows: 10 min at 94 °C, followed by 40 cycles of 30 s at 94 °C, 30 s at 49 °C, and 30 s at 72 °C, before being held for 7 min at 72 °C. All fragments were subjected to gel electrophoresis on 2% agarose gels containing ethidium bromide, before isolation for pyrosequencing.

##### Pyrosequencing

Primers were drawn to anneal adjacent to five methylated bases of the LHR-B segment and LHR-C segment of the *CR1* gene (Figure S2): methS0PYRO (5′–TTT-TAT-TTT-TTG-TTT-TTA-GG–3′), methS1PYRO (5′–GGT-TAT-TTA-TTT-GTT-GAA-TGT-ATT-T–3′), and methS2PYRO (5′–ATG-TAT-TTT-TTA-GGG-TAA-TGT-TGT–3′). The biotinylated PCR product (40 µL) was immobilized onto 4 µL of streptavidin-coated Sepharose beads (>1.2 nmol binding capacity; Amersham Pharmacia Biotech, Uppsala, Sweden) in 40 µL of binding buffer (10 mM Tris-HCl (pH 7.6), 2 M NaCl, 1 mM EDTA, and 0.1% Tween 20) on a shaker (1400 rpm) at room temperature for 10 min. PCR products immobilized on beads were transferred to a 96-well filter plate (Millipore, Molsheim, France) and vacuum-dried. Single-stranded DNA was obtained by adding 50 mL of denaturation solution (0.2 M NaOH) for 1 min. The immobilized strand was washed twice with 150 mL of washing buffer (10 mM Tris-acetate (pH 7.6), re-suspended in 40 µL of annealing buffer (20 mM Tris-acetate (pH 7.6) and 2 mM magnesium acetate), and transferred into wells of PSQ 96 Plate Low (40-0010, Qiagen, Courtaboeuf, France) containing 15 pmol of sequencing primer in a volume of 40 µL of annealing buffer. The plate was heated at 81 °C for 2 min. Real-time pyrosequencing was performed at 28 °C in an automated 96-well pyrosequencer using PSQ SNP 96, with enzymes and substrate (Pyrosequencing AB, Uppsala, Sweden), with cyclic dispensation of nucleotides. The computer analysis was based on an algorithm that compared the height of the different peaks and the base number of the polymorphic fragment.

#### 4.3.5. Determination of the *CR1* Length Genetic Polymorphisms Using High-Resolution Melting PCR (HRM-PCR)

The *CR1* length polymorphism was determined at the genetic level using HRM, as described previously [47]. Original primers (CN3: 5′–GGCCTTAGACTTCTCCTGC–3′ and CN3re: 5′–GTTGACAAATTGGCGGCTTCG–3′) were synthesized by Eurogentec (Seraing, Belgium). PCR was performed in a total volume of 20 µL, using 10 µL of 2× LightCycler 480 High-Resolution Melting Master Kit (Roche, Meylan, France), 1 µL of 300 nM forward primer, 1 µL of 300 nM reverse primer, and 10 ng of DNA. PCR was performed on a 96-well thermal cycler (Veriti; Applied Biosystems, Ontario, Canada) using an amplification protocol of one cycle at 95 °C for 10 min, followed by 45 cycles at 95 °C for 10 s, 62 °C for 15 s, and 72 °C for 20 s. The HRM of the amplicons using the LightCycler 480 System (Roche) displayed the melting-curve profiles corresponding to the four *CR1* length polymorphisms.

### 4.4. Quantification of sCR1 Using ELISA

Blood samples obtained in dry tubes were centrifuged at 1200× *g* for 10 min. Serum was aliquoted then frozen at −20 °C. An anti-sCR1 ELISA kit (USCN Life Science Inc., Houston, TX, USA) was used according to the manufacturer’s instructions. Serum was diluted 1/10, and the minimal dose of detectable sCR1 (sensitivity) was 0.124 ng/mL. The detection range was 0.312–20 ng/mL. All samples and standards were measured in duplicate, and the means were used for statistical analyses.

### 4.5. Statistical Analyses

Quantitative variables are presented as mean ± standard deviation (m ± SD), and qualitative variables as numbers (percentage). Univariate analysis was performed using comparison of means (Student’s *t*, Mann-Whitney *U*, or Kruskal-Wallis tests, or ANOVA), comparison of percentages (chi-squared or Fisher’s exact test), or correlation (Pearson correlation coefficient, *r*), as appropriate. Paired tests (Student’s paired *t*-test or the Wilcoxon test) were used for comparisons within groups, and unpaired tests for comparisons between groups. Bonferroni’s correction was applied to comparisons within groups and between groups for each genotype polymorphism (*Hin*dIII and Q981H), to control the alpha error due to the risk of inflation from multiple testing. Assuming five comparisons, *p*-values < 0.01 were considered significant for these tests. Agreement between the *Hin*dIII and Q981H genotypes was measured using the weighted Kappa coefficient. Multivariable analysis was performed to identify factors independently associated with AD. A logistic regression model was constructed using the stepwise method after adjustment for onset/inclusion age and other potential confounders (sex, *APOE*-ε4 genotype, and comorbidities). The interaction between *APOE* and *CR1* was also tested. The threshold for entry into and exit from the model was *p* < 0.20. The goodness-of-fit of the model was tested using the Hosmer-Lemeshow test. The results are presented as odds ratios (ORs) with 95% confidence intervals (CIs). A *p*-value < 0.05 was considered statistically significant. All analyses were performed using the SAS software (version 9.4; SAS Institute, Inc., Cary, NC, USA).

The association between density and methylation was explored using multivariate linear regression. Univariate linear regressions were performed between density and each covariate separately. Manual descending stepwise analysis was performed with methylation-related variables and all variables with a *p*-value strictly less than 0.20. AD was forced during the selection. The selection was complete when the *p*-value of all variables (except AD) was strictly less than 0.05. The overall *p*-values of qualitative variables were calculated using a likelihood ratio test. Mean methylation was calculated as the mean of the five methylation sites whenever the results of at least two methylation sites were available.

The variables selected for the stepwise procedure were density polymorphisms Q981H and *Hin*dIII, AD, age, *APOE*-ε4, methylation sites 1 to 5, and mean methylation (Table S3). No interaction was introduced into the model (Table S4). The final model included AD, age, density polymorphism *Hin*dIII, and the second methylation site. A *p*-value < 0.05 was considered statistically significant.

## 5. Conclusions

Our data (i) confirm the link between the long *CR1* isoform and AD; (ii) show that the long CR1 isoform, despite exhibiting more C3b/C4b binding sites per molecule, is less frequently expressed than the other *CR1* isoforms, probably through a higher methylation level; (iii) show that an acquired decrease in CR1/E in association with a higher level of sCR1 was observed in AD patients compared to controls; (iv) rather than an increase in complement downregulation and immune complexes or deposit removal initially inferred from the presence of the long CR1 isoform, which exhibits one additional C3b/C4b binding site per molecule, a less effective CR1 cleaning ability pattern in AD emerges progressively. This hypothesis opens new avenues for therapeutic research [12,48].

## 6. Patents

Rachid Mahmoudi, Aymric Kisserli, and Jacques HM Cohen are the inventors of a patent owned by the University of Reims Champagne-Ardenne (URCA) (patent number WO 2015166194).

## Figures and Tables

**Table 1:** Demographic and clinical characteristics of the study sample.

Variable	AD Patients (*n* = 100)	Controls (*n* = 87)	*p*
Age (years)	81.5 ± 7.2	74.3 ± 6.3	<10^−4^
Female sex	66 (66.0%)	50 (57.4%)	0.23
*APOE*-ε4+ (*n* = 73)	48 (48.0%)	25 (28.74%)	0.0071
Living at home	88 (88.0%)	83 (95.4%)	0.73
Comorbidities (Charlson)	1.31 ± 1.26	1.12 ± 1.02	0.27
Level of dependence			
IADL	4.81 ± 2.57	7.83 ± 0.86	<10^−4^
ADL	5.38 ± 1.0	5.95 ± 0.25	<10^−4^
Cognitive status			
MMSE	19.2 ± 5.3	28.8 ± 1.3	<10^−4^
AD stage			
Mild (MMSE ≥ 21)	42 (42.00%)	–	–
Moderate (MMSE 10–20)	55 (55.00%)	–	–
Severe (MMSE < 10)	3 (3.00%)	–	–

Notes: AD = Alzheimer’s disease; *n* = number of subjects; *APOE*-ε4+ = subject with at least one *APOE*-ε4 allele; IADL = instrumental activities of daily living, values range from 0 (completely dependent) to 8 (completely independent); ADL = activities of daily living, values range from 0 (completely dependent) to 6 (completely independent); MMSE = Mini-Mental State Examination, scores range from 0 to 30, whereby higher scores correspond to better cognitive status.

**Table 2:** Distribution of *CR1* density and length polymorphisms among AD patients and controls.

*CR1* Polymorphisms			Subjects		
			All (*n* = 187), %	AD Patients (*n* = 100), %	Controls (*n* = 87), %
Density polymorphisms	*HindIII*	HH	114 (61.0)	59 (59.0)	55 (63.2)
		HL	65 (34.8)	37 (37.0)	28 (32.2)
		LL	8 (4.3)	4 (4.0)	4 (4.6)
	Q981H	QQ	118 (63.1)	62 (62.0)	56 (64.4)
		QH	60 (32.1)	35 (35.0)	25 (28.7)
		HH	9 (4.8)	3 (3.0%)	6 (6.9)
Length polymorphisms	CR1*1 CR1*1		126 (67.4)	63 (63.0)	63 (72.4)
	CR1*1 CR1*1 + CR1*1 CR1*3		128 (68.5)	65 (65.0)	63 (72.4)
	CR1*1 CR1*2		48 (25.7)	28 (28.0)	20 (23.0)
	CR1*2 CR1*2		10 (5.4)	6 (6.0	4 (4.6)
	CR1*1 CR1*3		2 (1.1)	2 (2.0)	0 (0)
	CR1*1 CR1*2 + CR1*2 CR1*2		58 (31.0)	34 (34.0)	24 (27.6)
	CR1*2 CR1*4		1 (0.5)	1 (1.0)	0 (0)
	CR1*1 CR1*2 + CR1*2 CR1*2 + CR1*2 CR1*4		59 (31.6)	35 (35.0)	24 (27.6)
	CR1*2 CR1*2 + CR1*2 CR1*4		11 (5.9)	7 (7.0)	4 (4.6)

Notes: AD = Alzheimer’s disease; *CR1* = complement receptor 1, where numbers following asterisk denote an isoform; HH = individuals homozygous for the H allele (*Hin*dIII polymorphism); HL = individuals heterozygous for the *Hin*dIII polymorphism; LL = individuals homozygous for the L allele (*Hin*dIII polymorphism); QQ = individuals homozygous for the Q allele (Q981H polymorphism); QH = individuals heterozygous for the Q981H polymorphism; HH = individuals homozygous for the H allele (Q981H polymorphism).

**Table 3:** Multivariate analysis of factors associated with density.

Variable	Unit	Estimate	95% CIs	*p*
Alzheimer’s disease	–	−63.14	−144.89, 18.6	0.129
Age	1	−6.51	−11.79, −1.23	0.016
Density polymorphism *Hin*dIII (reference: HH)				<0.001
HL	–	−311.67	−389.16, −234.17	
LL	–	−569.51	−740.02, −398.99	
2nd methylation site	1%	−6.68	−12.37, −0.99	0.022

Notes: 95% CIs = 95% confidence intervals.

**Table 4:** Multivariate analysis of factors independently associated with Alzheimer’s disease at different levels of variation in the explanatory variables.

Variable	Unit	OR	95% CIs	*p*
Age (years)	1	1.182	1.118, 1.260	<0.0001
Sex (female)	–	2.605	1.172, 6.050	0.0215
*APOE*-ε4+	–	4.745	2.152, 11.199	0.0002
Density (number of CR1 antigenic sites per erythrocyte)	30	0.936	0.894, 0.975	0.0025
	100	0.801	0.689, 0.920	0.0025
	200	0.641	0.475, 0.847	0.0025
	400	0.411	0.225, 0.718	0.0025
	500	0.329	0.155, 0.661	0.0025
Density polymorphism Q981H (Q vs. HH)	–	12.416	1.603, 112.155	0.0193
Serum level of soluble CR1 (ng/mL)	1	1.032	1.013, 1.054	0.0015
	10	1.369	1.139, 1.685	0.0015
	20	1.874	1.298, 2.840	0.0015
	30	2.565	1.479, 4.787	0.0015
	40	3.512	1.684, 8.068	0.0015
	50	4.807	1.919, 13.597	0.0015

Notes: CR1 = complement receptor 1; OR = odds ratio; 95% CIs = 95% confidence intervals.

## Supplementary Materials

Supplementary materials can be found at http://www.mdpi.com/1422-0067/19/8/2175/s1. Figure S1: Comparison of the mean number of complement receptor 1 per erythrocyte (CR1/E) and soluble CR1 (sCR1) in AD patients according to AD stage. Box plots of CR1/E are shown. The upper and lower limits of the boxes, and the middle line across the boxes indicate the 75th and 25th percentiles, and the median, respectively. The upper and lower horizontal bars indicate the maximum and minimum values, respectively. A Student’s *t*-test was used for normally distributed variables; * *p* = 0.034. (a) Comparison of the mean number of CR1/E in AD patients according to the stage of AD. (b) Comparison of the level of sCR1 in AD patients according to the stage of AD stage; Figure S2: DNA sequence alignment of the high-resolution melting PCR (HRM-PCR) amplicons, and positions of the methylated sites according to the genomic reference sequence (NG 007481.1) corresponding to the long allele of *CR1* (CR1*2). Two areas of the long homologous region B (LHR-B) of *CR1* are amplified at positions 48743 to 48960 and 67300 to 67517 corresponding to the LHR-B segments, and one area is amplified at position 84358 to 84574 corresponding to the LHR-C segment. The positions of the five methylated sites (SM1, SM2, SM3, SM4, and SM5) are framed in color. In blue, SM1 is located at positions 48858, 67415, and 84472, SM3 is located at positions 48919, 67476, and 84533, SM4 is located at positions 48925, 67482, and 84539, and SM5 is located at positions 48939, 67496, and 84553. In orange, SM2 is located at positions 48895 and 67452, but is missing at position 84509; Table S1: Univariate linear regression between CR1/E density and MMSE score as a quantitative variable (values 0–30) and as a qualitative variable in two classes (Mild: 21 to 30 vs. moderate/severe: 0 to 20); Table S2: Univariate linear regression between SCR1 rate and MMSE score, as a quantitative variable (values 0–30) and as a qualitative variable in two classes (Mild: 21 to 30 vs. moderate/severe: 0 to 20); Table S3: Univariate analysis of density and covariates, ordered by *p*-value; Table S4: Tests of interaction.

## Abbreviations

AD	Alzheimer’s disease
AIDS	Acquired immunodeficiency syndrome
APOE	Apolipoprotein E
CR1	Complement component (C3b/C4b) receptor 1
GWAS	Genome-wide association studies
HRM	High-resolution melting
LHR	Long homologous repeat
SCR	Short consensus repeat
sCR1	Soluble form of CR1
SNP	Single-nucleotide polymorphism
