# Supplementary material for: Inherited and Acquired Decrease in Complement Receptor 1 (CR1) Density on Red Blood Cells Associated with High Levels of Soluble CR1 in Alzheimer’s Disease
Source: Int J Mol Sci. 2018 Jul 25;19(8):2175. doi: 10.3390/ijms19082175 (PMC6121509; doi:10.3390/ijms19082175)
Supplement: Supplementary file 1 [file ijms-19-02175-s001.pdf]

Supplementary figures

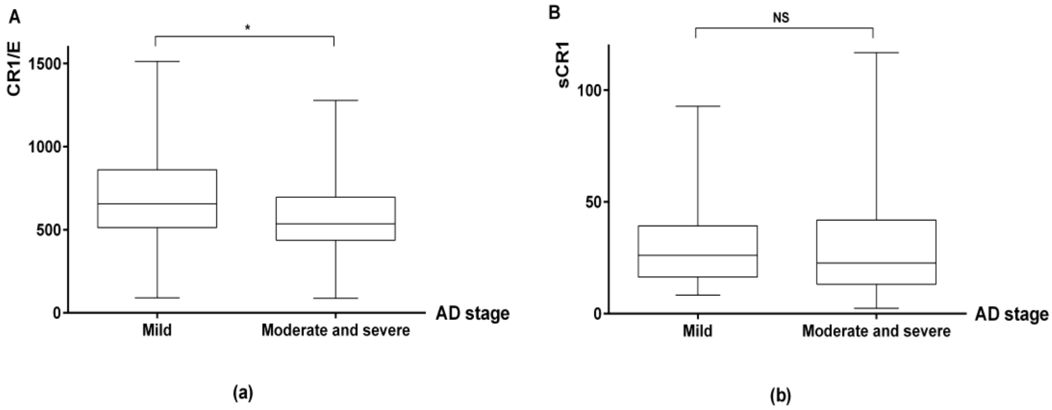

Figure S1. Comparison of the mean number of complement receptor 1 per erythrocyte (CR1/E) and soluble CR1 (sCR1) in AD patients according to stage of AD. Box plots of CR1/E are shown. The upper and lower limits of the boxes, and the middle line across the boxes indicate the 75th and 25th percentiles, and the median, respectively. The upper and lower horizontal bars indicate the maximum and minimum values, respectively. A Student's *t*-test was used for normally distributed variables; *p = 0.034. **(a)** Comparison of the mean number of CR1/E in AD patients according to the stage of AD. **(b)** Comparison of the level of sCR1 in AD patients according to the stage of AD.

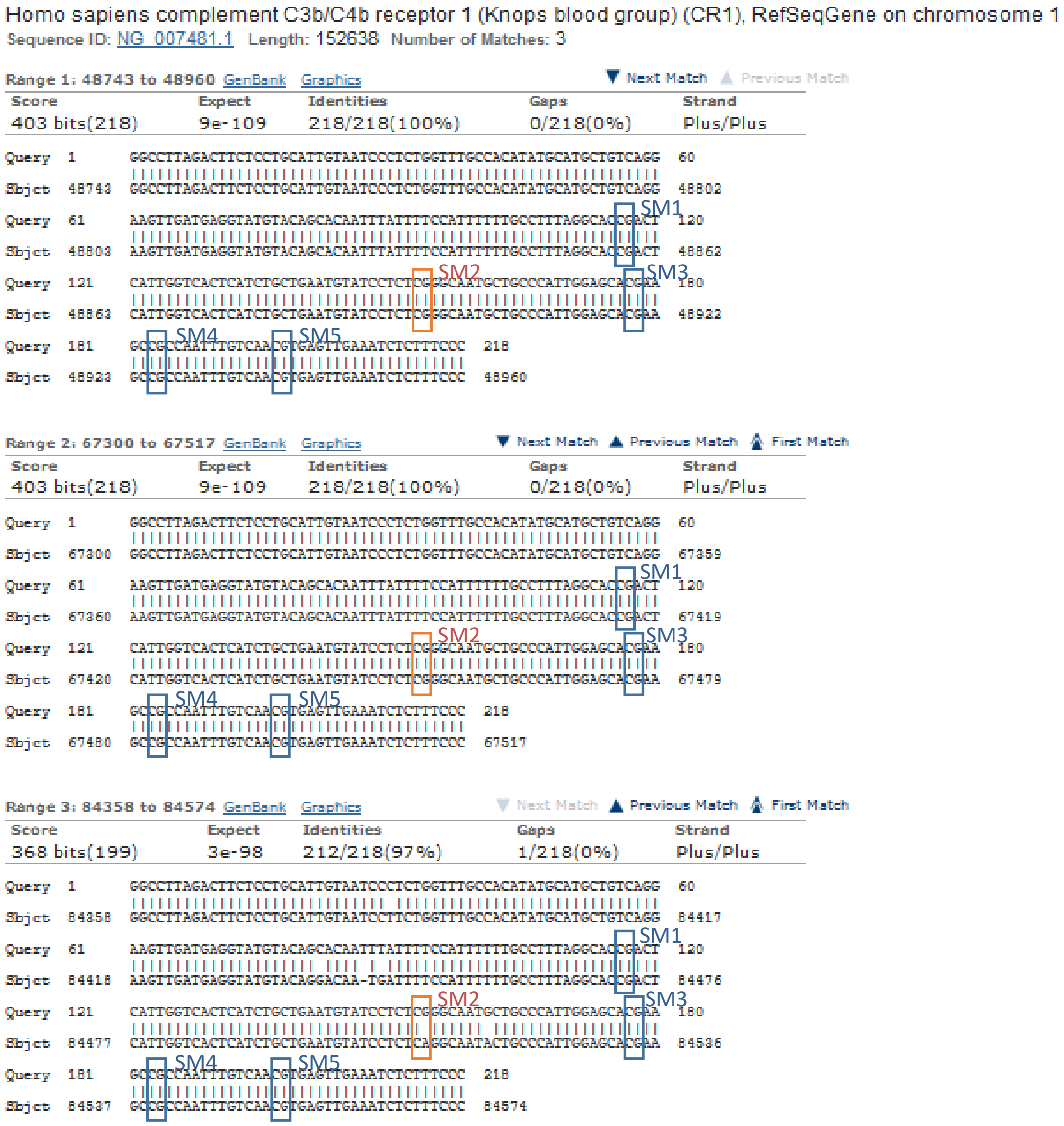

Figure S2. DNA sequence alignment of the high-resolution melting PCR (HRM-PCR) amplicons, and positions of the methylated sites according to the genomic reference sequence (NG 007481.1) corresponding to the long allele of *CR1* (CR1*2). Two areas of the long homologous region B (LHR-B) of *CR1* are amplified at positions 48743 to 48960 and 67300 to 67517, corresponding to the LHR-B segments, and one area is amplified at position 84358 to 84574, corresponding to the LHR-C segment. The positions of the five methylated sites (SM1, SM2, SM3, SM4, and SM5) are framed in color. In blue, SM1 is located at positions 48858, 67415, and 84472, SM3 is located at positions 48919, 67476, and 84533, SM4 is located at positions 48925, 67482, and 84539, and SM5 is located at positions 48939, 67496, and 84553. In orange, SM2 is located at positions 48895 and 67452, but is missing at position 84509.

Supplementary tables

Table S1 Univariate linear regression between CR1/E density and MMSE score as a quantitative variable (values 0–30) and as a qualitative variable in two classes (Mild: 21 to 30 vs. moderate/severe: 0 to 20).

Variable	Unit	Estimate	95% CIs	*p*
MMSE score	1	10.09	−0.01, 20.19	0.0503
MMSE score (reference: mild)	–	−115.08	−221.78, −8.39	0.035

Table S2 Univariate linear regression between SCR1 rate and MMSE score, as a quantitative variable (values 0–30) and as a qualitative variable in two classes (Mild: 21 to 30 vs. moderate/severe: 0 to 20).

Variable	Unit	Estimate	95% CIs	*p*
MMSE score	1	0.00	−0.88, 0.88	0.995
MMSE score (reference: mild)	–	−1.12	−10.45, 8.21	0.812

Table S3 Univariate analysis of density and covariates, ordered by *p*-value.

Variable	Unit	Estimate	95% CIs	*p*
Density polymorphism Q981H (reference: HH)	–			<0.001
HQ	–	146.844	−25.121, 318.81	
QQ	–	444.839	278.477, 611.2	
Density polymorphism *Hin*dIII (reference: HH)	–			<0.001
HL	–	−289.341	−363.063, −215.62	
LL	–	−530.712	−704.195, −357.229	
Alzheimer's disease	–	−110.591	−192.73, −28.453	0.009
Age	1	−3.809	−9.201, 1.584	0.165
APOE-ε4+	–	56.432	−28.745, 141.609	0.193
Length polymorphism (reference: CR1*1)	–			0.201
CR1*1 CR1*2	–	−105.815	−203.176, −8.455	
CR1*2	–	−92.002	−277.965, 93.961	
CR1*2 CR1*4	–	−290.902	−859.469, 277.665	
CR1*3 CR1*1	–	−95.902	−499.495, 307.691	
HRM (reference: EF)	–			0.281
F	–	93.79	−310.839, 498.419	
FS	–	0.042	−409.692, 409.775	
S	–	3.9	−435.872, 443.672	
T	–	−195	−890.341, 500.341	
4th methylation site	1%	3.742	−3.308, 10.791	0.296
1st methylation site	1%	−2.276	−6.868, 2.317	0.329
Sex (reference: male)	–	−29.151	−115.062, 56.76	0.504
3st methylation site	1%	−1.356	−6.203, 3.49	0.581
Mean methylation	1%	−1.678	−9.066, 5.71	0.655
2nd methylation site	1%	0.886	−5.948, 7.721	0.798
5st methylation site	1%	0.483	−5.587, 6.553	0.875

CR1 = complement receptor 1; 95% CIs = 95% confidence intervals.

Table S4 Tests of interaction.

Interactions	p
Length polymorphism × *APOE4*	0.406
Length polymorphism × Alzheimer's disease	0.263
APOE4 × Alzheimer's disease	0.344
Age × Alzheimer's disease	0.477
